# Correction: Survival Analysis of Adult Tuberculosis Disease

**DOI:** 10.1371/journal.pone.0118013

**Published:** 2015-02-10

**Authors:** The PLOS ONE Staff

The second author's name is spelled incorrectly. The correct name is: Zubair Kabir. The correct citation is: Ajagbe OB, Kabir Z, O'Connor T (2014) Survival Analysis of Adult Tuberculosis Disease. PLoS ONE 9(11): e112838. doi:10.1371/journal.pone.0112838
